# Do we know the place of systemic corticosteroids in infectious diseases?

**DOI:** 10.1186/1471-2334-13-S1-O23

**Published:** 2013-12-16

**Authors:** Adriana Hristea, Raluca Jipa, Oana Streinu-Cercel

**Affiliations:** 1 Carol Davila University of Medicine and Pharmacy, Bucharest, Romania; 2 National Institute for Infectious Diseases “Prof. Dr. Matei Balş”, Bucharest, Romania

## Background

Corticosteroids are drugs that can reduce the inflammation caused by infection, used as an adjunct to antimicrobial drugs to improve the outcome in various infectious diseases, but their role is controversial. Research on the use of corticosteroids in addition to antimicrobials has had conflicting results. Corticosteroids are also known to have certain adverse effects.

## Methods and results

We have studied the Cochrane systematic reviews addressing the systemic use of corticosteroids in:

• acute bacterial meningitis (to examine the effect versus placebo on mortality, hearing loss and neurological sequelae in participants of all ages); 24 randomized controlled trials (RCTs) enrolling 4041 patients;

• tuberculous meningitis (to examine the effect on death or disabling residual neurological deficit); 7 RCTs involving 1140 people;

• infectious mononucleosis (to assess efficacy and safety of steroids for symptom control); 4 trials that compared the effectiveness of a steroid for short-term symptom control to a placebo and 3 other trials that used a combination of steroid and an antiviral drug, involving 362 participants;

• acute sinusitis (to assess the effectiveness of systemic corticosteroids in relieving symptoms); 4 RCTs with a total of 1008 adult participants;

• acute pharyngitis (to assess the clinical benefit and safety of corticosteroids for symptoms of sore throat in adults and children) 8 trials involving 743 participants (369 children and 374 adults);

• acute pneumonia (to assess efficacy and safety of corticosteroids), 6 studies including 437 participants;

• croup in children (to determine the effect of glucocorticoids), 38 studies were included, enrolling 4299 patients;

• acute viral bronchiolitis in children (to evaluate the efficacy and safety of systemic and inhaled glucocorticoids), 17 trials with 2596 participants;

• severe sepsis and septic shock (to examine the effects of corticosteroids on death at one month in sepsis), 20 studies involving 2384 patients;

• tuberculous pleural effusion (to evaluate the effects of adding corticosteroids to drug regimens), 6 trials with 633 participants.

## Conclusion

The systematic review and meta-analysis facilitates an interpretation of variable results from different RCTs and is the first step in making evidence based medicine recommendations regarding corticosteroid use.

